# Chronic Post-Concussion Neurocognitive Deficits. II. Relationship with Persistent Symptoms

**DOI:** 10.3389/fnhum.2016.00045

**Published:** 2016-02-15

**Authors:** Jun Maruta, Lisa A. Spielman, Brett B. Yarusi, Yushi Wang, Jonathan M. Silver, Jamshid Ghajar

**Affiliations:** ^1^Brain Trauma FoundationNew York, NY, USA; ^2^Department of Psychiatry, New York University School of MedicineNew York, NY, USA; ^3^Department of Neurosurgery, Stanford UniversityStanford, CA, USA

**Keywords:** attention networks, mild traumatic brain injury, neuropsychology, post-concussion syndrome, predictive visual tracking

## Abstract

Individuals who sustain a concussion may continue to experience problems long after their injury. However, it has been postulated in the literature that the relationship between a concussive injury and persistent complaints attributed to it is mediated largely by the development of symptoms associated with posttraumatic stress disorder (PTSD) and depression. We sought to characterize cognitive deficits of adult patients who had persistent symptoms after a concussion and determine whether the original injury retains associations with these deficits after accounting for the developed symptoms that overlap with PTSD and depression. We compared the results of neurocognitive testing from 33 patients of both genders aged 18–55 at 3 months to 5 years post-injury with those from 140 control subjects. Statistical comparisons revealed that patients generally produced accurate responses on reaction time-based tests, but with reduced efficiency. On visual tracking, patients increased gaze position error variability following an attention demanding task, an effect that may reflect greater fatigability. When neurocognitive performance was examined in the context of demographic- and symptom-related variables, the original injury retained associations with reduced performance at a statistically significant level. For some patients, reduced cognitive efficiency and fatigability may represent key elements of interference when interacting with the environment, leading to varied paths of recovery after a concussion. Poor recovery may be better understood when these deficits are taken into consideration.

## Introduction

Following a concussion, the expectation of a full functional recovery within 7–10 days may be realized for most people (McCrory et al., 2013), but others continue to experience symptoms for an extended period of time (Kushner, 1998; Ryan and Warden, 2003; McMahon et al., 2014). The cluster of somatic, affective, and cognitive symptoms that commonly present together in these individuals has been recognized as postconcussive syndrome (World Health Organization, 1992; Alexander, 1995; Ryan and Warden, 2003). Although the term is widely accepted, this labeling has flaws since the symptoms that comprise postconcussive syndrome may not share a singular etiology and may evolve and conjoin over a period of time post-injury in nonspecific ways, possibly influenced by premorbid factors (King, 2003; Ryan and Warden, 2003; Ettenhofer and Barry, 2012; Silver, 2014).

In fact, the symptoms that persist after a concussion, including sadness, anxiety, sleep disturbance, irritability, concentration difficulties, and fatigue, are not specific to concussion (Iverson and Lange, 2003; Stein and McAllister, 2009; Ettenhofer and Barry, 2012; Cassidy et al., 2014). It has been postulated in the literature that the relationship between a concussive injury and persistent complaints attributed to it is mediated largely by the development of symptoms associated with posttraumatic stress disorder (PTSD) and depression (Hoge et al., 2008; Vasterling et al., 2012). Still, given that PTSD and depression are more likely to occur after a concussion than after other traumatic injuries (Hoge et al., 2008; Bryant et al., 2010), the original brain insult unmistakably contributes to the ensuing symptoms. There are also symptoms that appear more frequently after a concussion than after other injuries (Laborey et al., 2014). Thus, it is likely that the development of comorbid conditions incompletely accounts for persistent symptoms after a concussion.

Directly associating the original concussive injury with developed symptoms is difficult because the acute injury may not be followed by a chronic injury detectable by neuroimaging. In the absence of such evidence, the extent to which the original concussive injury may associate with the deficits after accounting for non-specific symptoms is unclear. We report in a companion paper a cohort of patients with persistent symptoms after a concussion in whom we failed to reveal anatomical indication of chronic injury using diffusion tensor imaging but nonetheless found some degree of cognitive deficits (Maruta et al., 2016). These cognitive deficits may be explained as well by the patients' self-ratings of symptoms as whether they had experienced a concussion (Hoge et al., 2008; Vasterling et al., 2012). The goal of the present study was to further characterize cognitive deficits in this cohort and to determine whether the original concussive injury retains significant associations with these deficits after accounting for the developed symptoms including those which overlap with PTSD and depression.

## Materials and methods

### Subject enrollment

Our research protocol was approved by the Weill Cornell Medical College Institutional Review Board and by the United States Department of the Army Human Research Protection Office. Patients with persistent symptoms after a concussion and control subjects were recruited via flyers posted on college campuses and at community centers throughout the New York City area. Patients were also recruited via referrals from health professionals. Recruitment information for both groups was made available on the Brain Trauma Foundation website and in the newsletters of other local brain injury organizations.

Recruitment was limited to male or female individuals 18–55 years of age and with at least 12 years of education. A trained research assistant administered a structured telephone screening of potential subjects that included medical, psychiatric, and substance use history questionnaires. Prospective control subjects completed the Conners' Adult Attention-deficit/hyperactivity disorder (ADHD) Rating Scales-Self-Report: Short Version (CAARS-S:S; Pearson, San Antonio, TX) and the Brain Injury Screening Questionnaire (BISQ; Gordon et al., 2000) at the time of screening, whereas patients completed these questionnaires at the time of testing. The test-retest reliability of CAARS-S:S is reportedly excellent although its specifics are unavailable (Conners et al., 1999). Three-month test-retest reliability of the BISQ measured with Cohen's kappa has been reported as 0.62 (Topolovec-Vranic et al., 2014). Patients additionally completed a modified Head Injury Symptoms Checklist (HISC; McLean et al., 1984) at the time of screening.

The BISQ was used to screen for any unidentified previous concussions in prospective control subjects and included self-reports of symptoms that are found to be common to traumatic brain injury. The BISQ was also used to classify patients according to the likelihood that their symptoms were related to concussion, posttraumatic amnesia (PTA), and loss of consciousness (LOC). Also completed at the time of testing were the Center for Epidemiological Studies—Depression Scale (CES-D; Radloff, 1977), the PTSD Checklist—Civilian Version (PCL-C; National Center for PTSD, US Department of Veterans Affairs), and the Wechsler Test of Adult Reading (WTAR; Pearson). Neither the CES-D nor the PCL-C is designed to measure a stable construct, but a 2-week test-retest correlation for the CES-D has been reported to be 0.51 (Radloff, 1977), and that for the PCL-C, 0.68 (Ruggiero et al., 2003). The WTAR was used with intent to estimate the subject's (premorbid) full-scale IQ (Green et al., 2008). Test retest correlations of 0.90–0.94 varying by age have been reported (PsychCorp, 2001).

To be considered for the patient group, a subject must have had: persistent problems believed to result from an isolated concussive head injury that occurred between 90 days and 5 years prior to the date of neurocognitive testing; documented medical attention at the time of injury; PTA at the time of injury; a complete BISQ; and if an LOC occurred, it did not exceed 24 h in the period following the injury. To be considered for the control group, a subject must have had a T-score less than 75 on the CAARS-S:S, a score <16 on the CES-D, and a negative BISQ outcome. Global exclusion criteria included: a history of gross vision or hearing problems; a history of a substance abuse; a history of a neurological or psychiatric disorder; general anesthesia within the 14 days prior to neurocognitive testing; current use of a psychotropic medication; and current pregnancy. An additional exclusion criterion in regard to past head injuries applied to both groups but differed between them. For the control group, any history of a confirmed concussive head injury or BISQ-identified injury was exclusionary, whereas for the patient group a history of prior concussive head injury was exclusionary only if it resulted in an emergency department visit that required conventional neuroimaging, seizures, or other medical problems.

Subjects deemed eligible to participate underwent testing at the Citigroup Biomedical Imaging Center at Weill Cornell Medical College. The administration of the full neurocognitive testing battery required ~2.5 h. During this period, participants were allowed brief rests as needed. Written informed consent was obtained immediately prior to the testing session and all subjects were monetarily compensated for their time. We recruited 147 subjects for the control group and 48 subjects for the patient group. We further reviewed each subject's participation eligibility following data collection (see Section Results).

### Reaction time-based cognitive assessments

The Automated Neuropsychological Assessment Metrics Version 4 (ANAM4) includes a library of computerized measures of cognitive performance (Reeves et al., 2007). The six performance tests were, in the order of administration: Simple Reaction Time (SRT), Code Substitution (CDS), Procedural Reaction Time (PRO), Mathematical Processing (MTH), Matching To Sample (M2S), and Code Substitution Delayed (CDD). A second administration of SRT (SR2), designed to measure performance under fatigue, was included at the end, ~20 min after the first administration. These tests, varying in complexity and in the composition of cognitive functions they drew on, were included in the battery for their presumed sensitivity to cognitive deficits commonly associated with head trauma (Eonta et al., 2011). Among a number of output measures, we utilized percent correct, throughput, and median reaction time (RT, measured in ms) of correct responses. The throughput metric is defined as the number of correct responses per minute, and thus is a useful metric for overall cognitive efficiency (Reeves et al., 2007). The median RT for correct responses is also a good metric of processing efficiency and elucidates a slightly different construct, because the metric considers only successful responses and because the median corrects for skew in the distribution of RTs more effectively than the mean (Carpenter, 1981).

In addition to the cognitive performance tests of the ANAM4 battery, we utilized the mean score of the Fatigue subscale of the Mood Affect Scale. Scores on this scale range from 0 to 6, with a higher score indicating increased fatigue. The self-assessment was made near the beginning of the testing sequence within the ANAM4 battery.

The Attention Network Test (ANT) is a computerized tool that combines elements of cued attention and flanker tasks to measure the efficiency of the neurocognitive networks involved in alerting, orienting, and conflict (executive control) attention, derived from facilitating or distracting effects of precue and target combinations (Fan et al., 2002, 2005). The overall accuracy across cue conditions and the overall processing efficiency, measured as the median of raw RTs (in ms) for accurate responses across cue conditions, were also recorded.

### Visual tracking

We measured subjects' eye movements while they tracked a predictably moving target. The details of the methods were described previously (Maruta et al., 2013). Briefly, subjects performed a circular visual tracking task on a video-based eye tracker integrated with stimulus-presentation (EyeLink 1000, SR Research Ltd., Mississauga, Ontario, Canada). The stimulus was presented on a 120 Hz LCD monitor (SyncMaster 2233RZ, Samsung, Seoul, South Korea). The stimulus consisted of a target that moved clockwise on a black background along a circular path with a radius of 10° at 0.4 Hz, at a constant speed of 25.1°/s. The task was performed in a normally lit room while subjects sat with their head stabilized by a chin-head rest. The visual acuity of each subject was confirmed to be normal or corrected-to-normal prior to testing.

The circular visual tracking task was given twice (Maruta et al., 2014b). The first trial (Trial 1) took place at the beginning in the sequence of neurocognitive assessment. The second trial (Trial 2) took place after an ~20-min interval during which subjects completed the ANT, an intensive attention-related task (described above). Each visual tracking trial, which included a practice run, a calibration procedure, and recorded runs, took approximately 5 min.

To characterize the stability of the gaze on the target, we evaluated the variability of gaze position error in degrees of visual angle along axes orthogonal (radial) and parallel (tangential) to target movement (standard deviation of radial and tangential errors—SDRE, SDTE). We also computed the mean phase error to characterize the central tendency of gaze position relative to the target, and the horizontal and vertical smooth pursuit velocity gains (H and V gains), which were the ratios between smooth pursuit eye velocity and target velocity. SDTE and mean phase error provided indications for temporal precision and accuracy of tracking (Maruta et al., 2013), while SDRE may increase with decreased vigilance (Maruta et al., 2014a,b; Tong et al., 2014).

### Analytic plan

Descriptive statistics (means and standard deviations for continuous variables, frequencies for categorical variables) were calculated for all measures. Between-group differences on all demographic characteristics were examined using chi-squared tests or between-group *t*-tests. Any demographic characteristic found to be significantly different between groups was examined for its association with neurocognitive performance metrics, and any found to be significant would have been entered as a covariate in subsequent analyses; however, none met that criterion. Between group differences on symptom-related variables from the CAARS-S:S, CES-D, PCL-C, and ANAM4 Fatigue scales, and neurocognitive performance metrics were examined using *t*-tests. For all between-group *t*-tests, Levene's test for equality of variances was examined. When comparisons were found to have significant heterogeneity of variances, the *t*-test for unequal variances with pooled standard deviations and degrees of freedom was applied. Findings are presented with a statistical significance defined by the alpha level of 0.05, and without correction for multiple comparisons because of the exploratory nature of the study.

To examine the effects that were directly or indirectly associated with concussive injury on the neurocognitive performance metrics, and to take into account the contributions of demographic- and symptom-related variables, a series of stepwise multiple linear regressions was performed. By this procedure, demographic- and symptom-related metrics that explicitly contributed to explaining group differences in the neurocognitive performance metrics were chosen. The neurocognitive performance metrics served as the dependent variables, and demographic- and symptom-related metrics served as the independent variables. Ethnicity was binary coded into white/Asian vs. all others based on the finding that the only significant differences on any metrics followed this grouping pattern. Income was omitted due to the large amount of missing data—a typical pattern when relying on self-report. Education is an important element of socioeconomic status (Diemer et al., 2013) and was included in the models. Since the CAARS-S:S subscale scores are highly interdependent, only one subscale was included in the models. We chose the inattention-memory problems subscale since this subscale showed the largest effect size of the difference of means between patient and control groups. For each model, group was force-entered as a binary code at the first step to examine the proportion of variance explained by group membership alone. Then it was removed and the demographic- and symptom-related variables along with group were examined for forward stepwise entry, with entry criteria set at *p* < 0.05 and removal criteria set at *p* > 0.10. This approach allowed us to determine: (a) whether group, by itself, accounted for a significant proportion of variance in outcome; (b) whether demographic- and symptom-related variables accounted for a significant proportion of variance in outcome, in the absence of group; and (c) whether group either remained significant, or emerged as significant in the context of the additional variables.

One patient did not undergo the WTAR because it was used as part of the evaluation at the referring medical facility. Two control subjects failed to make a sufficient number of correct responses on the ANT, which resulted in incomplete scoring of the test for these subjects. Technical errors accounted for missing test scores for one control subject on Trial 1 of the visual tracking test. This paper reports the results of analyses based on all observed data without regard for the missing data. In addition, visual tracking data from the control group were examined for outliers identified as lying outside ±3 SDs of the distribution. To allow us to fully characterize deviations from normal performance, these values were coded as missing only in the control group but not in the patient group. At most two outliers were identified in any of the visual tracking metrics.

## Results

Thirty-three subjects (aged 18–55, 16 male) met inclusion criteria for the patient group, and 140 subjects (aged 19–55, 67 male) met inclusion criteria for the control group, for a total of 173 subjects for the study. On average, patients were 1.6 years post-concussive injury, ranging from ~4 months to 4.5 years. Just over 60% indicated an LOC associated with their concussion, with the majority indicating LOC of <20 min. The 13 patients who did not have LOC experienced a period of PTA or a feeling of being “dazed and confused” for periods ranging from <5 min up to 24 h. Nearly all patients (32 out of 33) experienced a period of PTA associated with their concussion. Most (91%) endorsed cognitive, affective, and physical symptoms on the BISQ that had at least some probability of being associated with their concussion. On the modified HISC, all patients endorsed acquiring at least one symptom after the injury that did not exist prior to the injury or worsening of a previously existing symptom after the injury.

The patient and control groups did not differ significantly on chi-squared tests on gender or income, or on *t*-tests on age or WTAR-estimated IQ (Table 1). There was a significant group difference on ethnicity, with greater numbers of African American, Asian, and Hispanic subjects in the control group. There was also a significant group difference in years of education, with the control group reporting nearly 2 more years on average than the patient group. The groups differed significantly on *t*-tests on the CES-D, the PCL-C, the CAARS-S:S, and the ANAM4 Fatigue scale such that the patient group endorsed higher levels of symptoms on all four measures.

**Table 1:** **Demographic- and symptom-related group characteristics**.

	**Patient**		**Control**		***p***		
**GENDER**							
Male	16 (48.5%)		67 (47.9%)		≅1.00		
Female	17 (51.5%)		73 (52.1%)				
**ETHNICITY**							
White	30 (90.1%)		88 (62.9%)		0.040		
African American	1 (3%)		20 (14.3%)				
Asian	1 (3%)		16 (11.4%)				
Hispanic	1 (3%)		8 (5.7%)				
Unknown	0 (0%)		8 (5.7%)				
**INCOME ($ PER ANNUM)**							
0–10K	1		13		0.19		
10–20K	2		7				
20–40K	2		26				
40–60K	2		24				
60–100K	9		25				
>100K	9		35				
(missing response)	10		8				
	**Mean (*****SD*****)**	***N***	**Mean (*****SD*****)**	***N***	**|*****t*****|**	***df***	***p***
Age (years)	34.9 (14.0)	33	36.6 (10.6)	140	0.63	41.1	0.53
Education (years)	15.2 (2.5)	33	17.0 (2.4)	140	3.72	171	0.000
**CAARS-S:S**							
Inattention-memory problems	60.3 (13.8)	33	42.8 (6.7)	139	7.08	35.6	0.000
Hyperactivity-restlessness	51.3 (11.2)	33	43.3 (7.1)	139	3.94	38.3	0.000
Impulsivity-emotional liability	50.5 (12.6)	33	41.9 (4.9)	139	3.87	34.7	0.000
Problems with self-concept	51.2 (11.5)	33	40.8 (5.5)	139	5.04	35.5	0.000
ADHD index	55.1 (11.8)	33	40.8 (6.2)	139	6.73	36.3	0.000
CES-D	19.8 (13.6)	33	6.1 (5.2)	140	5.69	34.2	0.000
PCL-C	39.1 (15.8)	33	21.0 (5.2)	140	6.51	33.6	0.000
WTAR full-scale IQ	109.5 (8.0)	32	110.1 (7.7)	140	0.39	170	0.70

On the ANAM4 cognitive performance tests, the groups did not differ on *t*-tests on CDS or CDD. Significant differences were observed in processing efficiency (throughput or median RT for correct responses) on SRT, PRO, MTH, M2S, and SR2, with patients performing more poorly than control subjects (Table 2). A difference was not observed in the percent correct index on any of the tests. There was an improvement in median RT between SRT and SR2 for both groups, which was consistent with the known practice effect (Eonta et al., 2011). However, the group difference was observed only for SR2. On the ANT, the groups did not differ on *t*-tests on the measures of the specific attention components that this test is designed to separate out, or on the accuracy of responses. The groups differed on the grand mean effect, a measure of overall processing efficiency, with patients performing more poorly than controls subjects. On the visual tracking test, the groups differed on *t*-tests on H gain for Trial 1, with patients performing more poorly than control subjects. For Trial 2, the groups differed on SDRE and SDTE, again with patients performing more poorly than control subjects.

**Table 2:** **Neurocognitive performance characteristics**.

	**Patient**		**Control**				
	**Mean (*SD*)**	***N***	**Mean (*SD*)**	***N***	**|*t*|**	***df***	***p***
**ANAM4**							
SRT							
Throughput	202.8 (41.4)	33	218.4 (34.0)	140	2.26	171	0.025
SR2							
Median RT correct	274.1 (60.6)	33	251.7 (33.2)	140	2.05	36.6	0.047
Throughput	197.8 (42.0)	33	221.7 (30.3)	140	3.75	171	0.000
PRO							
Median RT correct	588.0 (129.0)	33	537.0 (70.0)	140	2.2	36.6	0.035
Throughput	92.0 (20.1)	33	104.6 (14.0)	140	3.44	39.6	0.001
MTH							
Median RT correct	2390.3 (719.2)	33	2045.5 (553.9)	140	3.03	171	0.003
Throughput	23.3 (6.9)	33	27.1 (6.7)	140	2.87	171	0.005
M2S							
Median RT correct	1827.1 (690.7)	33	1567.3 (482.7)	140	2.05	39.7	0.047
**ANT**							
Grand mean effect	655.7 (166.5)	33	572.2 (74.5)	138	2.81	35.1	0.008
**VISUAL TRACKING**							
Trial 1							
H gain	0.90 (0.06)	33	0.93 (0.06)	137	2.15	168	0.033
Trial 2							
SDRE	0.90 (0.44)	33	0.69 (0.20)	138	2.71	35.3	0.010
SDTE	1.22 (0.71)	33	0.95 (0.46)	139	2.10	38.7	0.042

Next, neurocognitive performance was examined in the context of demographic- and symptom-related variables in a series of stepwise multiple linear regressions. Table 3 shows Spearman correlations of cognitive performance test metrics to PTSD and depression symptoms scores. Some of the correlations of performance metrics to depression symptoms reached statistical significance while none of those to PTSD symptoms did. However, in general, weak to moderate correlations (>0.1), indicating increased information processing time and decreased efficiency with larger symptom scores, were often found.

**Table 3:** **Spearman correlations of cognitive performance test metrics to PTSD and depression symptoms scores**.

	**PCL-C**		**CES-D**	
	***rho***	***p***	***rho***	***p***
**ANAM4**				
SRT				
Median RT correct	0.20	0.26	0.09	0.62
Throughput	−0.16	0.37	−0.04	0.84
SR2				
Median RT correct	0.10	0.57	0.17	0.34
Throughput	−0.25	0.16	−**0.36**	**0.039**
CDS				
Median RT correct	0.31	0.074	0.34	0.054
Percent correct	0.12	0.59	0.07	0.70
Throughput	−0.32	0.068	−0.33	0.065
PRO				
Median RT correct	0.30	0.085	**0.37**	**0.033**
Percent correct	−0.06	0.72	−0.12	0.51
Throughput	−0.33	0.065	−**0.38**	**0.031**
MTH				
Median RT correct	0.22	0.215	0.22	0.22
Percent correct	0.09	0.62	0.10	0.572
Throughput	−0.17	0.36	−0.16	0.36
M2S				
Median RT correct	0.32	0.070	**0.45**	**0.0090**
Percent correct	0.14	0.44	0.07	0.69
Throughput	−0.21	0.25	−**0.35**	**0.043**
CDD				
Median RT correct	0.32	0.068	0.27	0.13
Percent correct	−0.02	0.89	−0.15	0.40
Throughput	−0.27	0.13	−0.21	0.23
**ANT**				
Alerting effect	−0.16	0.38	−0.21	0.25
Orienting effect	−0.28	0.115	−0.06	0.75
Conflict effect	−0.01	0.97	0.21	0.25
Accuracy	−0.09	0.62	−0.07	0.69
Grand mean effect	−0.01	0.96	0.20	0.27

*Bold typeface indicates p < 0.05. N = 33*.

Tables 4, 5 list performance metrics for which group either remained a significant contributor to the variance in the outcome or emerged as such only in the context of the additional variables. Performance metrics for which group was not a significant contributor to the variance in the outcome at any step or for which demographic- and symptom-related variables accounted for significant portion of the outcome in the absence of group were omitted. The *p*-value in the forced predictor column indicates whether group by itself accounted for a significant proportion of the variance. The contributions of group and additional variables in the final selected model are shown in the stepwise predictor columns. For the ANAM4 subtests, group retained a significant association for at least one of the SRT, CDS, PRO, and CDD indices, but not for any of the MTH, M2S, or SR2 indices (Table 4). For the ANT indices, group emerged as a significant associate of the conflict effect, and accuracy, in addition to the grand mean effect as observed in the between-group *t*-tests. For the visual tracking test, group also emerged as a significant associate of SDRE and SDTE for Trial 1, in addition to H gain. For Trial 2, group was a significant associate of SDRE, SDTE, as it was in the between-group *t*-tests, but also mean phase error and H gain (Table 5).

**Table 4:** **Stepwise multiple linear regressions examining reaction time-based task performance in the context of demographic- and symptom-related variables**.

**Dependent variable**		**Predictor**							**Model statistic**		
		**Forced**	**Stepwise**								
		**Group**	**Group**	**Age**	**Gender**	**Ethnicity**	**IQ**	**CES-D**	***R*^2^**	***F***	***p***
**ANAM4**											
SRT Throughput	*B*	−16.35	−16.26		−21.04		1.19		0.21	13.00	0.000
	|*t*|	2.33	2.53		4.00		3.49				
	*p*	0.021	0.012		0.000		0.001				
CDS Median RT correct	*B*	136.98	173.11	14.31		−125.99	−7.58		0.41	26.26	0.000
	|*t*|	2.32	3.65	8.50		2.46	2.93				
	*p*	0.021	0.000	0.000		0.015	0.004				
Throughput	*B*	−4.09	−5.50	−0.60		4.85	0.35		0.42	26.89	0.000
	|*t*|	1.69	2.85	8.70		2.33	3.35				
	*p*	0.094	0.005	0.000		0.021	0.001				
PRO Percent correct	*B*	−2.38	−2.38						0.055	8.92	0.003
	|*t*|	2.99	2.99								
	*p*	0.003	0.003								
Throughput	*B*	−12.83	−7.95	−0.45		7.56	0.36	−0.49	0.38	17.85	0.000
	|*t*|	4.21	2.47	4.69		2.66	2.52	3.56			
	*p*	0.000	0.014	0.000		0.009	0.013	0.001			
CDD Median RT correct	*B*	149.24	167.18	16.96			−9.25		0.34	26.09	0.000
	|*t*|	2.07	2.79	7.83			2.95				
	*p*	0.040	0.006	0.000			0.004				
**ANT**											
Conflict effect	*B*	29.06	29.14	0.96			−1.60		0.15	8.71	0.000
	|*t*|	2.80	2.94	2.68			3.09				
	*p*	0.006	0.004	0.008			0.002				
Accuracy	*B*	−1.36	−1.36						0.038	6.07	0.015
	|*t*|	2.46	2.46								
	*p*	0.003	0.003								
Grand mean effect	*B*	90.51	97.83	3.19	54.49				0.32	23.21	0.000
	|*t*|	4.55	5.53	4.96	3.78						
	*p*	0.000	0.000	0.000	0.000						

**Table 5:** **Stepwise multiple linear regressions examining visual tracking performance in the context of demographic- and symptom-related variables**.

**Dependent variable**		**Predictor**								**Model statistic**		
		**Forced**	**Stepwise**									
		**Group**	**Group**	**Age**	**Gender**	**Ethnicity**	**IQ**	**Education**	**Fatigue**	***R*^2^**	***F***	***p***
**TRIAL 1**												
SDRE	*B*	0.13	0.13							0.056	9.01	0.003
	|*t*|	3.00	3.00									
	*p*	0.003	0.003									
SDTE	*B*	0.15	0.20		0.19	−0.22				0.10	5.80	0.001
	|*t*|	1.64	2.19		2.68	2.45						
	*p*	0.10	0.030		0.008	0.016						
H gain	*B*	−0.03	−0.03		−0.03		0.00			0.13	7.42	0.000
	|*t*|	2.53	2.64		2.94		2.16					
	*p*	0.012	0.009		0.004		0.032					
**TRIAL 2**												
SDRE	*B*	0.25	0.20				−0.01		0.04	0.19	11.85	0.000
	|*t*|	4.82	3.71				2.55		2.21			
	*p*	0.000	0.000				0.012		0.029			
SDTE	*B*	0.35	0.33				−0.01			0.13	11.58	0.000
	|*t*|	3.73	3.68				−2.92					
	*p*	0.000	0.000				0.004					
Mean phase error	*B*	0.68	1.00				−0.44	0.18		0.10	5.84	0.001
	|*t*|	1.99	2.87				2.48	2.87				
	*p*	0.048	0.005				0.014	0.005				
H gain	*B*	−0.02	−0.03	−0.021		0.04				0.10	5.31	0.002
	|*t*|	1.66	2.23	2.08		2.74						
	*p*	0.10	0.027	0.039		0.027						

## Discussion

We found a variety of cognitive deficits in patients with persistent symptoms after a concussion even though these patients likely represented those with a concussive injury at the milder end of the spectrum (Maruta et al., 2016). Patients were generally found to produce accurate responses on RT-based tests, but with reduced information processing efficiency. On predictive visual tracking, patients' performance was generally similar to that of control subjects when they were tested initially, but when tested immediately after engagement with an attention demanding task, the variability of the gaze position around the target was greater in patients than in control subjects, indicating a greater task-related fatigability for patients. Isolated deficits in specific cognitive domains were not evident across different tests. When neurocognitive performance was examined in the context of demographic- and symptom-related variables, including but not limited to PTSD and depression, some of the test scores retained significant association with the original injury, while other test scores were more strongly associated with the developed symptoms or demographic variables. For still other test scores, effects of the original injury on neurocognitive performance emerged by accounting for symptoms and demographic variations. For example, the original injury was not found to affect Trial 1 SDTE, but it emerged as a significant association when gender and ethnicity were included in the model while the contribution of PTSD or depression symptom score to the variance was deemed non-significant. Our results are consistent with the notion that a reduction in cognitive performance may not be due to greater symptom report itself, but can be associated with the initial concussive injury (Dean and Sterr, 2013).

The results also suggest an important distinction between self-reported general fatigue state, and temporary, task-related or cognitive fatigue. The indication that patients were more susceptible to task-related fatigue followed the results of repeat testing of two objective measures—the visual tracking task and the simple visuo-manual reaction task of the ANAM4. We reported similar task-related fatigability effects in a cohort of patients with ADHD previously (Maruta et al., 2014b). The self-assessment of fatigue state further supported a general reduction of energy in patients. Importantly, however, although self-report of fatigue state was a characteristic more strongly associated with patients, it was not an explanation for their reduced cognitive performance.

The present findings should be validated with more closely matched patient and control groups since there were some demographic differences in our two groups. Although presently demographic variables were included in the stepwise multiple regression approach, an analytical tool should not be viewed as a replacement for having a good control group. A further limitation of this study was that the analysis of the outcome measures did not allow us to delineate the nature of the association between the presumed cognitive fatigability and the concussive injury. However, in light of our finding that the efficiency in information processing may be reduced in patients, fatigability may be explained as larger energy expenditure in generating comparable-to-normal responses. A parallel may be drawn with a study that revealed deficits under increased physiological stress of normobaric hypoxia in patients with a concussion history who were otherwise asymptomatic (Temme et al., 2013). Similarly, in a recent study, patients with persistent post-concussion symptoms were shown to have abnormal blood oxygen level-dependent activity in functional MRI during a visual tracking task although their tracking performance was normal (Astafiev et al., 2015). Thus, patients' neural circuits may have been altered but there was a functional compensation, only to reveal deficits latently under increased physiological or cognitive stress. Nevertheless, such neural circuit alteration may still be an indirect consequence of the concussive injury (Stein and McAllister, 2009; Rathbone et al., 2015), and further research is needed.

## Conclusion

The label of post-concussive syndrome itself may exemplify the implicit but pervasive assumption that there is a singular etiology for the symptoms that persist after a concussion. Such an assumption needs to be reconsidered. We posited that characterization of cognitive deficits should reveal insights into processes underlying these symptoms. In a cohort of patients with persistent symptoms after a concussion, we did not find isolated deficits in specific cognitive domains, but identified broad reduced information processing efficiency and fatigability. These deficits were found to be associated with the original concussive injury even after accounting for the developed symptoms. Reduced information processing efficiency and fatigability may represent key elements of interference with interacting with the environment for some patients, leading to varied paths of recovery after a concussion. Poor recovery may be better understood when these deficits are taken into consideration.

## Author contributions

JM and JG designed experiments and oversaw data collection and analysis. BY and YW contributed to neuropsychological testing and subject screening. JM, BY, YW, and LS contributed to data management. LS conducted statistical analyses. All authors contributed to the interpretation of data and to drafting and revising the work.

### Conflict of interest statement

JG is director of Sync-Think, Inc. and holds U.S. patent 7,384,399. JM holds stock option in Sync-Think. The authors declare that the research was conducted in the absence of any other commercial or financial relationships that could be construed as a potential conflict of interest. The reviewers, MV and TL, and handling Editor declared their shared affiliation, and the handling Editor states that the process nevertheless met the standards of a fair and objective review.

## Abbreviations

ADHD: Attention-deficit/hyperactivity disorder
ANAM4, Automated Neuropsychological Assessment Metrics Version 4 (SRT: Simple Reaction Time
CDS: Code Substitution
PRO: Procedural Reaction Time
MTH: Mathematical Processing
M2S: Matching To Sample
CDD: Code Substitution Delayed
SR2: second administration of SRT)
ANT: Attention Network Test
BISQ: Brain Injury Screening Questionnaire
CAARS-S = S: Conners' Adult ADHD Rating Scales-Self-Report = Short Version
CES-D: Center for Epidemiological Studies—Depression Scale
HISC: Head Injury Symptoms Checklist
LOC: loss of consciousness
PCL-C: PTSD Checklist—Civilian Version
PTA: posttraumatic amnesia
PTSD: posttraumatic stress disorder
RT: reaction time
SD: standard deviation
SDRE: SD of radial errors
SDTE: SD of tangential errors
WTAR: Wechsler Test of Adult Reading.
